# Starch-Based Functional Ingredients in Baking: A Review of Advances in Starch Derivatives, Quality Enhancement, and Reduction of Fermentable Oligosaccharides, Disaccharides, Monosaccharides, and Polyols

**DOI:** 10.3390/molecules31101709

**Published:** 2026-05-18

**Authors:** Eric Biney, Min Wang, Kit-Leong Cheong

**Affiliations:** 1 Guangdong Provincial Key Laboratory of Aquatic Product Processing and Safety, Guangdong Province Engineering Laboratory for Marine Biological Products, Guangdong Provincial Engineering Technology Research Centre of Seafood, Guangdong Provincial Engineering Technology Research Center of Prefabricated Seafood Processing and Quality Control, College of Food Science and Technology, Guangdong Ocean University, Zhanjiang 524088, China; ericbi1831@gmail.com; 2 College of Coastal Agriculture Sciences, Guangdong Ocean University, Zhanjiang 524088, China; wangmin@gdou.edu.cn

**Keywords:** starch, modified starch, hydrocolloids

## Abstract

Starch (native and modified) is a major polymer in baked goods, affecting dough rheology, crumb structure, and shelf life. This review covers recent advances in starch functionalization for baking, including chemical, physical, and enzymatic modifications. It further examines how other polysaccharides (e.g., gums and fibres) modulate starch-based systems, and finally addresses strategies to reduce fermentable oligosaccharides, disaccharides, monosaccharides, and polyols (FODMAPs) in bakery products. Emphasis is placed on novel technologies, trade-offs (prebiotic fiber vs. FODMAPs), and gaps needing future research.

## 1. Introduction

Baked goods are a beneficial source of daily energy, protein, and dietary fiber for many people around the world. As staples, they are good carriers of crucial nutrients that can improve the performance of technology and the quality of nutrition. Polysaccharides are among the most essential components of baked products. Their structural diversity, which includes both native and modified starches, non-starch cell wall polysaccharides, hydrocolloids, gums, and oligosaccharides, provides bakery systems with a range of functions [1]. They are utilized to enhance dough management, augment aeration, regulate moisture migration, inhibit staling, and prolong product shelf life. Simultaneously, polysaccharides affect nutritional outcomes by regulating glycemic response, enhancing dietary fiber intake, and serving as fermentable substrates for gut microbiota [2,3]. Nonetheless, several beneficial polysaccharides, particularly fructans and specific oligosaccharides such as inulin, are classified as fermentable oligosaccharides, disaccharides, monosaccharides, and polyols (FODMAPs). These may elicit symptoms in susceptible individuals and vulnerable populations, particularly when diet quality or nutritional adequacy is compromised by dietary restrictions or by nutritional risks, such as inflammatory bowel disease [4]. Consequently, the dual design issue confronting contemporary bakery science is to regulate the FODMAPs load while employing polysaccharides to achieve technological and health objectives. The annual demand for both conventional and functional foods derived from starch and its derivatives is increasing [5]. The non-food sectors are increasingly interested in this biopolymer due to concerns regarding the supply of petrochemical raw materials and environmental challenges associated with post-production waste recycling. Therefore, the quest for novel techniques that enhance the multifunctional properties of starch continues [6]. Previous focus was on advancements in both non-selective and selective functionalization of starch to produce products with ideal utility in the food sector (resistant starch (RS), dextrins, and maltodextrins) and non-food sectors (hydrophobic and oxidized starch). However, food texture, flavour, performance, and nutritional content are affected by its physical properties throughout preparation, heating, storage, and shelf life. Adding polysaccharides to food is considered a safe, effective, and successful modification process, alongside chemical, physical, and enzymatic methods. Nonetheless, there is a scarcity of studies on the influence of polysaccharides on the physical characteristics of foods.

Chemical manipulation has been shown to improve the performance of natural foods; nevertheless, it can be harmful to consumers and pollute the environment [7]. The use of modifying enzymes, though a good and long-lasting strategy, is limited by their specificity and selectivity, making it challenging to produce the desired product. Therefore, the addition in product development is proposed as the best, safest, and most effective modification approach. As a result, current research has investigated how polysaccharides influence stability, emulsifying and gelling capabilities, rheological and suspension properties, viscoelasticity, water retention capacity, and starch digestion and regeneration. The limitations of starch use, novel approaches, and implemented techniques to address processing challenges have garnered significant attention [8]. This review assesses the properties of starch and its versatility as a biopolymer, highly useful across many industrial applications.

## 2. Advances in Starch Derivatives and Their Functionality in Baked Goods

### 2.1. Classes of Starch Derivatives Relevant to Baking

**Figure 1:**
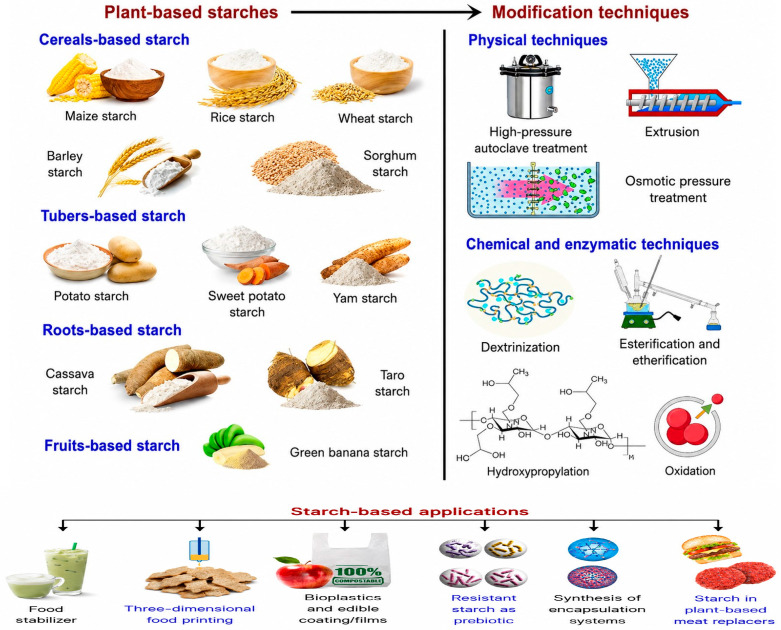
Conventional plant sources, methods for modification, and applications of starches. Reproduced from [9]. Copyright 2024, Environmental Chemistry Letters.

Physically modified (pregelatinized, annealed, heat-moisture treated), chemically modified (cross-linked, acetylated), enzyme-treated, and RS types (RS2, RS3, RS4) are examples of starch derivatives used in baking. Gelatinization, pasting, retrogradation, water binding, and interactions with gluten and non-gluten proteins are all altered differently by each class. Clean-label methods (physical/enzymatic modification) are becoming increasingly significant in the functional design of starch components. Simultaneously, chemical cross-linking remains crucial for applications requiring exceptional heat or shear stability [9]. The quality and functionality of baked goods are greatly influenced by the starch derivatives commonly used in them (Figure 1). For example, they can improve the nutritional, flavour, shelf-life, and textural profiles of breads, cookies, and crackers. Derivatized starches have already been shown to have several potential uses in baked goods, such as prebiotics, delivery methods, fat substitutes, and inks for 3D printing, enabling baked goods to be precisely customized and nutritious.

### 2.2. Mechanisms Affecting Baked Product Quality

The complex dynamic interactions between physicochemical transformations during processing and baking determine the quality of baked products, including volume, texture, crumb structure, colour, and shelf life [10]. The formation and stability of the aerated structure, phase transitions and microstructure setting, flavour and colour progression, and post-baking modifications influencing staling are the four main mechanistic pillars that make up baked product quality. Starch derivatives and polysaccharide-based ingredients act as functional modifiers via several mechanisms. The determination of the final sensory experience of baked products through ingredients and procedures is grounded in scientific concepts at the molecular and macrostructural levels, such as polymer science, colloidal chemistry, thermodynamics, and microbiology.

### 2.3. Water Binding and Dough Viscosity

**Figure 2:**
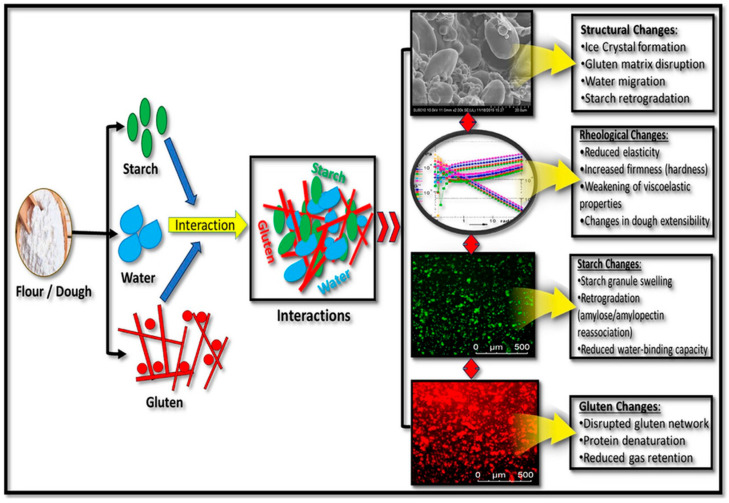
Schematic representation of the mechanisms that influence the quality of baked goods. Adopted from [11]. Copyright 2025, Comprehensive Reviews in Food Science and Food Safety.

Modified starches and other polysaccharides are hydrocolloids that can hold a lot of water. These make dough or batter hold more water by forming hydrogen bonds with water molecules, reducing the availability of free water for gluten formation (Figure 2). This affects dough viscosity and elasticity, which, in turn, affects gas cell stability during baking and fermentation. Cross-linked or hydroxypropylated starches may retain more water, resulting in a dough structure that is more stable and less prone to spoilage. Regression and crumb firming also slow water retention, and starches or hydrocolloids can stabilize the dough structure; they do not necessarily reduce susceptibility to spoilage.

### 2.4. Gluten Network Modification

**Figure 3:**
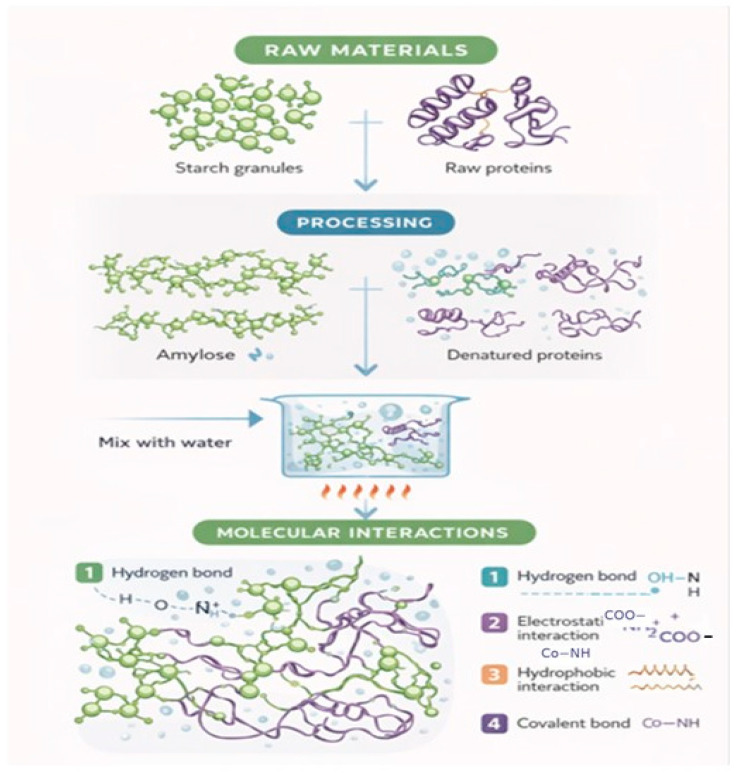
Starch and gluten work together to improve bread properties.

The gluten matrix strength depends on the type and quantity of polysaccharides. Non-starch polysaccharides, including guar gum, xanthan gum, and arabinoxylans, compete with gluten proteins for water, making it harder for gluten to absorb water and form networks. On the other hand, specific oxidized polysaccharides or altered starches may enhance interactions between proteins and polysaccharides, resulting in dough that is more elastic and capable of retaining greater volumes of gas. These structural changes affect bread volume, crumb porosity, and elasticity (Figure 3).

Starch granules serve as fillers within a three-dimensional protein matrix through mixing and the gradual absorption of water. During baking, starch and gluten proteins compete for water, thereby influencing starch gelatinization. As temperature increases, the microstructures of gluten proteins undergo alterations, exposing their hydrophobic regions, while starch helices denature, exposing their hydroxyl groups. Specifically, hydrogen bonding contributes to water retention and network stabilization, while hydrophobic interactions enhance protein aggregation and dough elasticity. These interactions collectively influence dough viscoelasticity, gas retention, crumb structure, and final bread texture during baking.

### 2.5. Gas Retention and Cell Structure Formation

During fermentation and initial baking, the rheological properties of the dough determine the extent of gas hold-up and expansion. To an extent, gas hold-up and expansion are critical determinants of bread volume, crumb structure, and texture. Poor gas retention leads to dense products, whereas optimal rheology ensures proper loaf expansion and desirable sensory properties. Starches with high paste viscosities strengthen the continuous phase and reduce coalescence, both of which help air cells [12]. A thicker dough polysaccharides mixture makes the interaction between gas and liquid more stable. This produces a refined and consistent crumb structure, as observed with the incorporation of methylcellulose and carboxymethyl cellulose (CMC) in both wheat and gluten-free formulations [13].

### 2.6. Starch Gelatinization and Pasting Behavior

**Figure 4:**
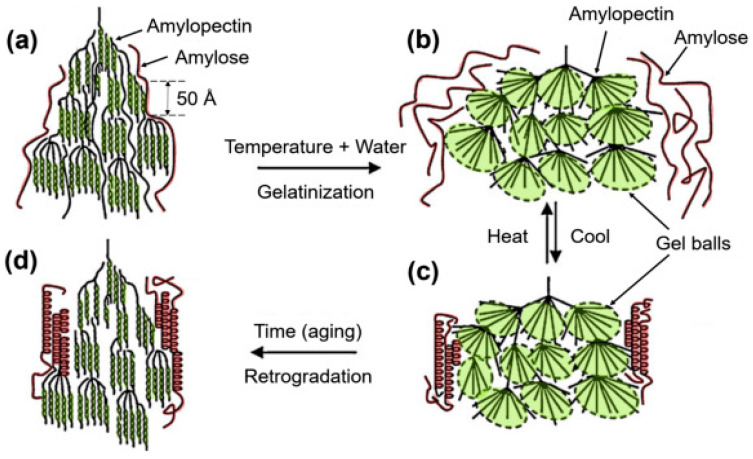
Gelatinization and retrogradation processes. (**a**) Starch granules remain intact; (**b**) Water absorption causes swelling, molecular segregation, and amylose loss to solution; (**c**) Cooling realigns amylose molecules, (**d**) Recrystallisation of Storage of amylopectin molecules [16] Copyright, 2020. Elsevier.

Native and modified starches are essential in crumb formation via gelatinization. The temperature and gelatinization rate determine water absorption and starch granule swelling, thereby influencing the final texture and moisture distribution [14]. Cross-linking can increase the gelatinization temperature by approximately 5–15 °C and reduce swelling power by 20–50%, depending on the degree of modification, resulting in more stable crumb structures after baking (Figure 4). Pregelatinized starches, in contrast, enhance batter viscosity before baking, which is advantageous for gluten-free recipes [15].

### 2.7. Retrogradation and Staling

**Figure 5:**
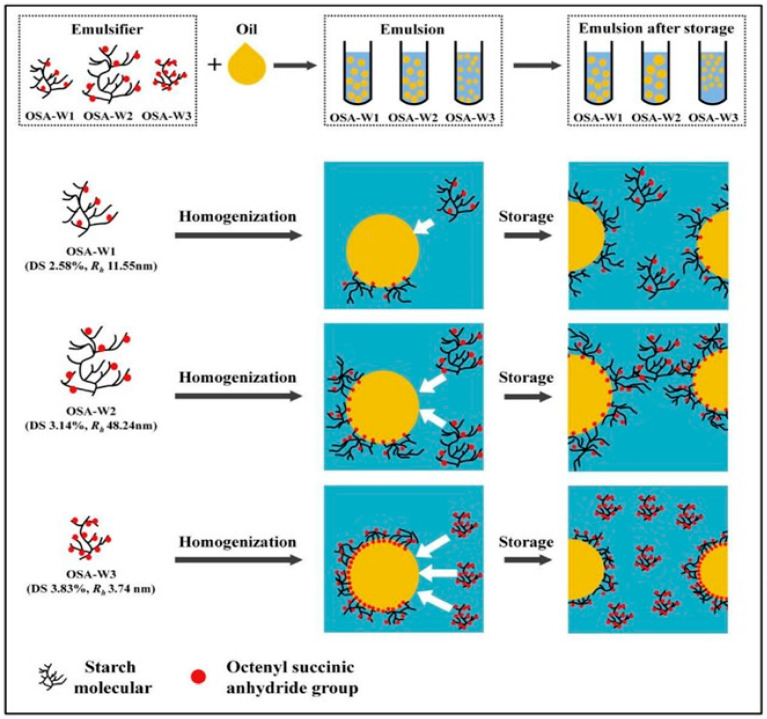
Schematic representation of the emulsification and stabilization mechanisms of OSA-starches with multi-scale architectures. Adapted from [20]. Copyright 2018, Elsevier.

Research conducted by Chen et al. [17] showed that starch retrogradation, mainly due to amylopectin recrystallisation, results in crumb firming during storage. Modified starches containing hydroxypropyl, acetyl, or octenyl succinate groups disrupt the alignment of amylopectin chains, thereby decreasing the rate and extent of retrogradation [18]. This mechanism extends the freshness of baked goods by preserving moisture and softness, and prolonging the shelf life of commercial bakery items. For example, certain modified starches and polysaccharides possess surface-active characteristics that enhance emulsification, such as octenyl succinic anhydride (OSA)-modified starch, acetylated starch, and hydroxypropyl starch, which are known to exhibit surface-active and emulsifying properties, as shown in Figure 5, making them more airy and larger [19]. These interactions yield a more refined crumb and improve mouthfeel and tenderness.

### 2.8. Crust Formation and Browning

**Figure 6:**
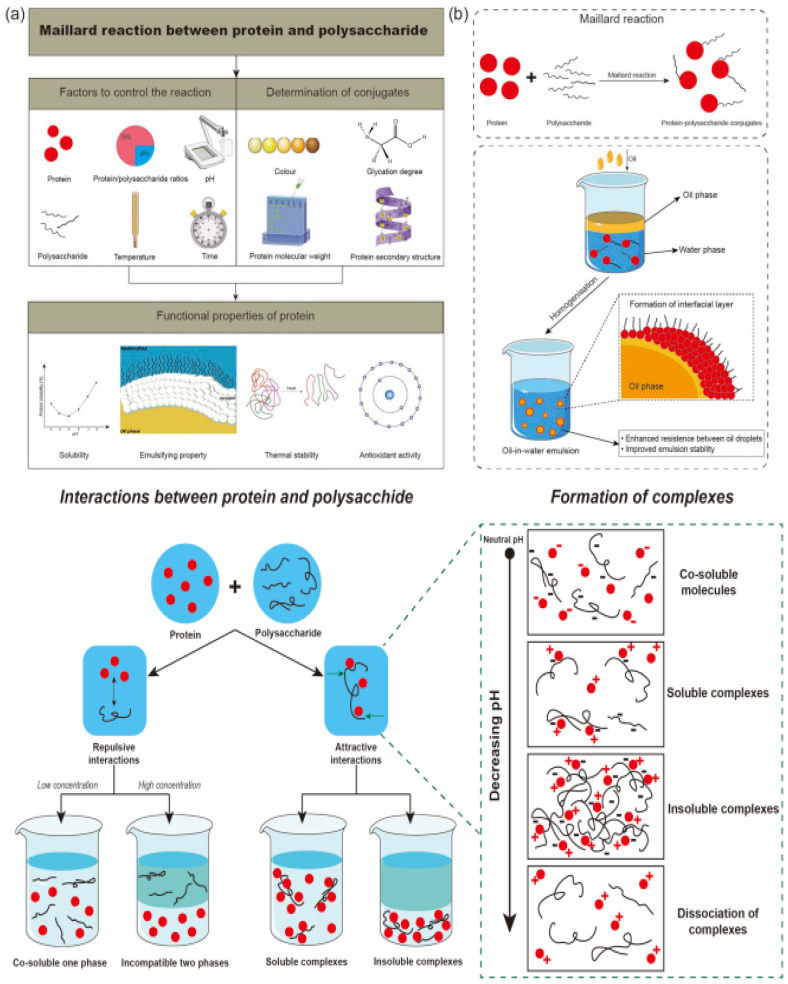
Maillard reaction involving a protein and polysaccharide: (**a**) impact of the Maillard reaction on the functional characteristics of the protein; (**b**) synthesis of protein polysaccharide conjugates and the mechanism of emulsion formation by these conjugates. The interaction between a protein and a polysaccharide in aqueous systems during complex formation is pH-dependent. Adapted from [21].

The water-binding capacity and thermal stability of polysaccharides affect heat transfer and moisture migration in baking. This is because hydroxyl (-OH), sulfate (-OSO_3_^−^), and acetyl groups influence water binding, viscosity, and thermal conductivity. These functional groups alter moisture diffusion and heat transfer by modifying matrix density and water mobility within the food system. Decreased surface moisture accelerates Maillard reactions and caramelization, influencing crust colour, crispness, and flavour. Moreover, polysaccharides can modify heat and mass transmission dynamics, resulting in more uniform crust and crumb development, as shown in Figure 6.

Natural starch exhibits numerous unfavorable characteristics, including thermal instability and a tendency to form opaque, fragile gels, which constrain its utility. Starch is frequently changed to address these constraints. This method improves favourable characteristics, such as paste clarity, gel strength, and adhesiveness, while mitigating disadvantages, such as retrogradation (the separation of water from the gel) and syneresis [22]. Contemporary starch modification methods are categorized into physical, chemical, or enzymatic procedures. Physical modification includes heat techniques (e.g., heat–moisture treatment, annealing, roasting) to alter molecular ratios and chain lengths, non-thermal techniques (e.g., high-pressure, irradiation), and mechanical techniques (e.g., milling, extrusion) [23]. Chemical modification entails grafting functional groups onto the starch backbone via hydrolysis, oxidation, or esterification to impart new properties (Table 1) [24]. Enzymatic modification provides precise control over molecular structure; however, it is limited to disintegration and does not enhance the granules’ swelling capacity [25].

## 3. Starch-Polysaccharide Interactions in Dough

**Figure 7:**
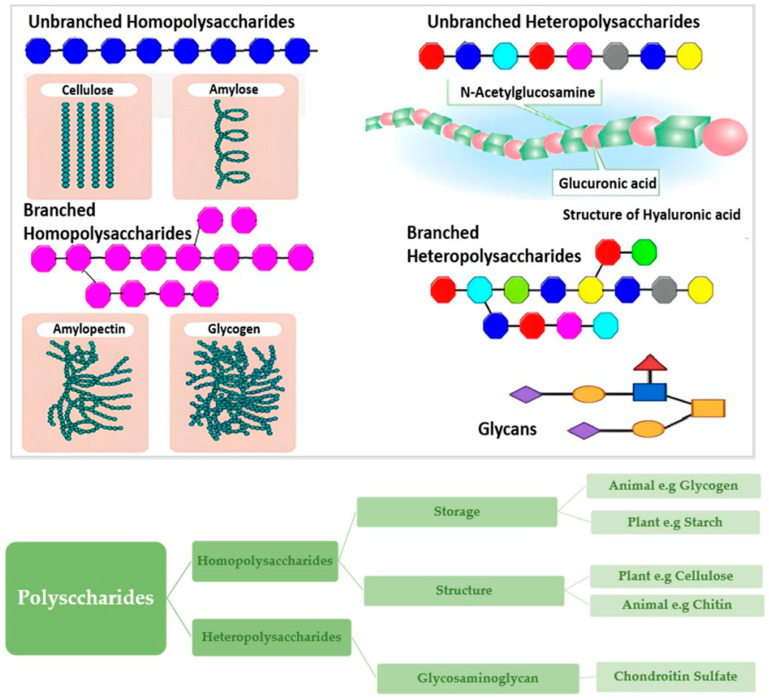
The various colours of monosaccharides. These include both branched and unbranched homopolysaccharides and heteropolysaccharides. Classifying polysaccharides according to their physiological characteristics and the specific monosaccharides utilised in their synthesis [33].

Polysaccharides are high-molecular-weight carbohydrates formed by monosaccharide units connected through glycosidic linkages [28]. They are found naturally in plants, animals, fungi, and microorganisms, making them one of the most prevalent types of biopolymers [29]. They are essential in food formulation, materials science, medication delivery, and tissue engineering due to their wide range of physicochemical properties and functional effects arising from their extensive structural diversity [30]. As depicted in Figure 7, polysaccharides are primarily used in food systems as thickeners, stabilisers, gelling agents, fat substitutes, and film formers. Numerous polysaccharides have physiological benefits beyond their technological applications, such as controlling gut microbiota, acting as prebiotics, lowering blood sugar levels, and regulating the immune system [31]. The classification of polysaccharides into representative classes and the discussion of their primary effects provide an extensive framework for comprehending the potential applications of polysaccharides in the food and health sciences [32].

To improve freeze-thaw stability, solubility, and gel strength, modified starches that have been physically, chemically, and enzymatically altered have been developed due to issues with retrogradation and syneresis. Starches are the primary source of digestible carbohydrates in the human diet, while RS fractions offer prebiotic benefits and assist in glycemic regulation. Marine algae-derived polysaccharides, such as alginates, carrageenans, and agar, are frequently used as gelling, thickening, and stabilizing agents. Alginates create ionotropic gels with calcium, carrageenans yield thermoreversible gels, and agar serves as a resilient gelling agent utilized in both culinary and microbiological applications [34]. These polysaccharides demonstrate significant bioactivities, including antiviral, antioxidant, and anti-inflammatory properties [35]. Fucoidans, sulfated polysaccharides, are found in the cell walls of brown algae (*Phaeophyceae*) such as *Fucus vesiculosus*, *Laminaria japonica*, *Undaria pinnatifida*, and *Ascophyllum nodosum*. As one of the most bioactive marine polysaccharides, they have been studied for their antioxidant, anticoagulant, antiviral, anticancer, and immunomodulatory properties due to their structural diversity and biocompatibility [35]. Fucoidans are popular in pharmaceuticals, nutraceuticals, and cosmeceuticals [36]. Bacterial polysaccharides, including xanthan, gellan, dextran, and pullulan, constitute a rapidly expanding class of functional biopolymers [37]. Xanthan gum is esteemed for its elevated viscosity and shear-thinning properties, gellan gum for its transparent, thermoreversible gels, and dextran for its prebiotic and medicinal uses.

Cellulose is an insoluble linear β-(1 → 4)-linked glucan that plays a fundamental structural role in plant cell walls as show in Table 2 [38]. Carboxymethyl cellulose (CMC), hydroxypropyl methylcellulose (HPMC), and methylcellulose are among its derivatives, widely used as stabilisers, emulsifiers, and fat substitutes. These altered celluloses have unique properties, such as thermogelation, that make them useful in low-fat recipes and gluten-free baking [39]. Hemicelluloses are diverse polysaccharides (e.g., xylans, mannans, arabinoxylans) that are linked with cellulose in the cell walls of plants. Their capacity to regulate water-binding, viscosity, and gas retention renders them significant in baked goods. Arabinoxylans specifically improve dough stability and demonstrate prebiotic properties [40].

Pectins are intricate polysaccharides abundant in galacturonic acid, predominantly derived from citrus peels and apple pomace. In response to sugar and acid (high-methoxyl pectins) or calcium ions (low-methoxyl pectins), they form gels. Pectins are widely used in jams, jellies, and dairy products, and they also decrease cholesterol and change the immune system [41,42]. Natural gums, including gum arabic, guar gum, and locust bean gum, are significant hydrocolloids characterized by their high water-binding ability. They work as emulsifiers, stabilisers, and thickening agents. Gums and mucilage serve critical functions in pharmaceuticals as tablet binders, drug carriers, and controlled-release excipients.

Furthermore, nanotechnology has improved its applications in environmental cleanup and drug delivery through hydrogels and mucilage-coated nanoparticles. Despite its potential, obstacles exist due to diversity in extraction and processing methods. Guar gum is used for its dietary fiber properties, particularly its role in regulating glycemic response. Gums are hydrophobic or hydrophilic polysaccharides with high molecular weights that produce gels or viscous solutions in appropriate solvents. Food, textiles, biomedicine, cosmetics, and pharmaceuticals use hydrocolloids, water-soluble gums. Their widespread use is due to their cost-effectiveness, accessibility, biocompatibility, chemical stability, non-toxicity, water-binding properties, gel-forming ability, and emulsion-stabilizing properties [35].

**Table 2:** An overview of the primary origins and chemical compositions of several plant-derived mucilages.

Plant Name	Botanical Name	Plant Part	Chemical Components	Reference
Chia	*Salvia hispanica* L.	Seed	D-xylose, D-glucose, D-glucuronic acid, galacturonic acid	[43]
Basil	*Ocimum basilicum* L.	Seed	Glucan, Xylan, and glucomannan	[44]
Psyllium	*Plantago ovata*	Seed	d-xylose, L-arabinose, and D-galacturonic acid	[45]
Flax	*Linum* *usitatissimum* L.	Seed	d-galacturonic acid, l-rhamnose, galactose, and d-xylose	[46]
Fenugreek	*Trigonella foenum-graecum* L.	Seed	polysaccharides, soluble & insoluble fiber	[47]
Cassia	*Cassia obtusifolia* L.	Seed	Mannose, galactose, and glucose	[48]
Cress	*Lepidium sativum*	Seed	Glucuronic acid, galacturonic acid, mannose, rhamnose, fructose, arabinose	[49]
Wild Sage	*Salvia macrosiphon*	Seed	Mannose, rhamnose, arabinose, galactose, glucose, and galactomannan	[50]
Qodume shirazi	*Alyssum homalocarpum*	Seed	Rhamnose and galactose	[51]
Balangu Shirazi	*Lallemantia royleana*	Seed	galactose, arabinose, rhamnose, xylose, and glucose	[52]
Quince	*Cydonia oblonga* Miller	Seed	Xylose, glucose, galactose, fructose	[53]
Qudome shahri	*Lepidium perfoliatum*	Seed	Rhamnose, xylose, arabinose, galactose, glucose	[54]
Okra	*Abelmoschus esculentus* L.	Bark	L-rhamnose, D-galactose, and L-galacturonic acid	[55]
Cactus	*Opuntia ficus-indica*	Leafy	arabinose, galactose, galacturonic acid, rhamnose, and xylose.	[56]
Malabar spinach	*Basella alba*	Leafy	galactose, arabinose, galacturonic acid, and rhamnose	[57]
Aloe vera	*Aloe vera Lepidium*	Leafy	glucomannans, amino acids, lipids, sterols, and vitamins	[58]
Asario	*Lepidium sativum*	Seed	mannose, arabinose, galacturonic acid, fructose, glucuronic acid, galactose, rhamnose, glucose	[59]
Hibiscus	*Hibiscus rosa-sinensis*	Bark	L-rhamnose, D-galactose, D-galacturonic acid, and D-glucuronic acid	[60]
Date pulm	*Phoenix dactylifera*	Bark	carbohydrates, pectin, cellulose, and starch.	[61]
Banana	*Musa paradisiaca*	Peel	flavanols, hydroxycinnamic acids, flavan-3-ols, and catecholamines. cellulose,	[62]
Orange	*Citrus reticulata* Blanco	Peel	cellulose, lignin, hemicellulose.	[63]
Shameplant	*Mimosa pudica*	Seed	xylose, glucose, and glucuronic acid	[64]

The rheological properties of dough are critical determinants of its ability to entrap and retain gases during fermentation and baking, hence impacting bread volume, crumb texture, and overall product quality [65]. Dough is a viscoelastic system in which gluten proteins provide the elastic framework, starch granules act as fillers, and polysaccharides control hydration, viscosity, and network interactions. The dough’s effectiveness throughout mixing, proving, and baking depends on the balance between elasticity and extensibility [66].

Water-soluble polysaccharides, such as inulin, guar gum, xanthan gum, or low-Dextrose maltodextrins, enhance dough viscosity and strength, preventing the coalescence and rupture of gas bubbles. These are instrumental in gluten-free doughs or matrices with weak gluten, as they retain water, preventing gas diffusion and stabilizing the foam [67]. From a rheological perspective, these additives often increase the storage modulus (G′), helping the dough maintain its shape during proving by making it more elastic. Too much viscosity in baking can make the dough more complicated to work with, harder to use machines, and less likely to rise [68]. Rheological experiments consistently demonstrate that the incorporation of polysaccharides increases the storage modulus (G′). This modification reduces the dough’s flexibility while enhancing its appearance. While a lack of extensibility may prevent the dough from expanding and cause gas cells to break too quickly, greater elasticity helps maintain the stability of bubbles throughout the proving process. Therefore, formulations must be improved to achieve the ideal dough viscoelasticity, sufficient collapse resistance, and sufficient oven spring extensibility [69].

The connection between dough rheology and gas retention extends beyond mixing and proving [70]. The dough matrix is further pressed by rapid gas expansion and steam production during baking. Bubble integrity and the formation of uniform crumbs are enhanced by polysaccharides that either increase the thickness of the film surrounding the bubbles or strengthen the gluten starch hydrocolloid composite network. On the other hand, uneven cell distribution, structural collapse, or sticky textures can occur if insoluble fibres aren’t well mixed or are too stiff.

Polysaccharides affect dough rheology and product texture by regulating water distribution, starch-gluten interactions, and air-cell stability. Hydrocolloids interacting with starch: guar gum and xanthan gum bind water and compete with starch/gluten, thereby altering starch gelatinization and dough viscosity [71]. Modified starches can enhance crumb elasticity and facilitate sliceability. When starch is hydrated and kneaded, the granules swell, allowing water to enter more easily. Molecules become more mobile over time (water and starch molecules can move around more easily). Excessive viscosity may hinder dough manipulation, resulting in inadequate machinability, an inconsistent crumb structure, or a sticky, dense texture [72].

Furthermore, insoluble polysaccharides (e.g., hemicelluloses formed from bran) might interfere with the gluten network, leading to rougher textures and less loaf volume unless supplemented with softening agents. That illustrates that HPMC made it easier to work with the dough by helping it hold its shape and control moisture. Bread becomes firmer as the HPMC content increases, indicating greater gas retention and softer bread. Adding HPMC also significantly increased the specific volume, resulting in larger, more stable loaves [73]. But too much xanthan gum prevented gas cells from forming, making the loaves denser. The formulations with the highest HPMC content lost the least moisture during baking, thereby remaining moist during heating. Adding more xanthan gum increased water activity (aw), indicating greater surface moisture.

On the other hand, HPMC and guar gum showed slight reductions, which could prolong product shelf life [74]. The colour property showed that as the levels of HPMC and guar gum increased, the lightness of the crust and the crumb (L*) increased. Nonetheless, as the levels of xanthan gum increased, this is likely due to more intense Maillard reactions and denser crumb formations [75]. Overall, HPMC had the most significant effect on bread quality, making it larger, softer, and better colored. Too much xanthan gum, on the other hand, had the opposite effect. In gluten-free bread systems, appropriate amounts of the three hydrocolloids are needed to achieve the desired balance of dough stability, texture, and appearance, as shown in Table 3 [76,77]. Xanthan gum is not a starch-based polysaccharide; its inclusion in this review is justified by its widespread use as a functional hydrocolloid in bakery systems, where it often serves as a benchmark or complementary ingredient to starch. Xanthan gum exhibits unique rheological properties, including high viscosity at low concentrations, shear-thinning behavior, and strong water-binding capacity [78].

Polysaccharides mainly help extend food shelf life by regulating moisture and preventing starch spoilage. The addition of gum maintains the crumb’s high-water activity, slowing the firming process during storage. Alginate, pectin, and carboxymethylcellulose can make protective matrices that stop moisture from moving between the crumb and the crust. They help prevent the texture from worsening [81]. Additionally, lipid oxidation in enriched breads is reduced because many polysaccharides prevent oxygen from passing through. A trade-off occurs when excessive moisture retention makes the crust too soft, leaving it less crisp than consumers prefer. If storage conditions are not ideal, high water-binding capacity may accelerate microbial degradation in severe cases. Although polysaccharides typically soften and make foods easier to chew, their impact on sensory quality must be carefully examined [82]. A normal amount of crumb feel makes items more moist, but too much can make them sticky or taste bad [83]. Water-retaining gums may block Maillard browning, making crusts lighter and the flavours of roasting or caramelizing less appealing to consumers. High-viscosity matrices can trap volatile molecules, making fresh bread smell and look less attractive. Bread is made chewier by some RS and fibres, which is good for artisan breads but bad for soft sandwich loaves [84].

Formulators must carefully select the type, concentration, and functional form (native versus modified) of polysaccharides to improve product quality [85]. Combining water-binding gums with emulsifiers or enzymes is an example of a combinatorial strategy that can enhance suppleness while eliminating sensory drawbacks. Positioning your product is very important. Consumers might be okay with whole-grain or “health-oriented” loaves that are thicker and chewier, but white bread needs to be as soft as possible and free of intense flavours [86]. The viscoelastic gluten network is essential for making dough, holding gas, and giving the finished product its texture; therefore, baking without gluten is hard. Polysaccharides are essential structural alternatives because they mimic gluten’s ability to hold water, stretch, and keep gas [81]. Due to their ability to bind water and thicken, hydrocolloids such as xanthan, guar, locust bean gum, and HPMC are primarily used to improve dough viscosity and stabilize gas cells during proofing and baking. These polymers make a pseudo-network when mixed with modified starches. That keeps the loaf’s volume and makes the crumb softer and airier. Because they can form films and cross-link, egg, soy, whey, and pea proteins enhance the polysaccharide starch matrix and strengthen the network [87]. When used together, xanthan guar or xanthan HPMC gives the bread a finer structure and helps it stay fresher than when used alone. They also keep the bread from getting soggy while still allowing it to taste delicious. Using cutting-edge technologies, gluten-free systems are made to work with enzymes. Transglutaminase-mediated covalent cross-links among protein substrates enhance network flexibility and strength. The use of tailored starches, such as resistant or chemically modified starches, in conjunction with transglutaminase improves dough manipulation and extends shelf life, as reported in [88]. Prebiotic fibres like inulin and -glucans have recently been added to gluten-free baking to meet consumer demand for healthier alternatives, providing structural and nutritional benefits [89].

## 4. FODMAPs in Bakery Systems: Sources, Health Implications, and Polysaccharide-Based Reduction Strategies

### 4.1. FODMAPs in Cereal and Bakery Ingredients

FODMAPs are short-chain carbohydrates that exhibit poor absorption in the small intestine and undergo fast fermentation in the colon, resulting in gas production and osmotic water influx into the lumen. In susceptible individuals, especially those with irritable bowel syndrome (IBS), this may result in bloating, stomach discomfort, diarrhea, or constipation. Cereal and bakery-based foods are significant dietary sources of FODMAPs, rendering them a primary focus in nutrition and food product development. Fructans are composed of β-(2 → 1) linked fructose units, typically featuring a terminal glucose [90]. Fructans are the most common FODMAPs in wheat-based breads and pastries used in baking systems. During dough fermentation, it provides fermentable substrates for yeast and lactic acid bacteria [91]. Fructans can be hydrolyzed during prolonged fermentation, as in sourdough, making the food easier to digest and lowering FODMAP levels. Incorporated as prebiotic fibres, derived from chicory root or Jerusalem artichoke, and occasionally occurring naturally in wheat. Functions in baking include enhancing water retention, improving the tenderness of crumbs, and substituting for fat to improve gut microbiota through bifidogenic actions [92].

FODMAPs, fast fermentation causes IBS symptoms, even though they are known to have prebiotic benefits [93]. Some examples include milk powder, whey protein, yoghurt or cream fillings, dairy-based garnishes, and lactose in baked products. It assists with Maillard browning and makes things taste better when they’re inside items. The primary problem is for individuals who do not produce enough lactase, though it is becoming increasingly important as more lactose-free products are introduced [94]. Legume flours such as soy, chickpeas, lentils, and peas are increasingly used in gluten-free or high-protein baked goods. Food and nutrition: Prebiotic potential similar to that of FOS that encourages beneficial gut microbiomes [95]. The functional component of legume flours enhances the nutritional profile, water absorption, and protein content. In sensitive individuals, fermentability can cause gastrointestinal discomfort [96]. Sugar-free cookies and chewing gum use polyols, which are sugar alcohols such as sorbitol, mannitol, maltitol, and xylitol, as well as bakery fillings. Certain fruits (apples, plums, cherries) utilized in baked goods also contain polyols. Roles within baking systems: Thickening agents and moisture-retaining substances. Decrease caloric content while preserving sweetness. FODMAP issue: Inadequate absorption and osmotic effects may induce bloating, gas, and diarrhea at moderate dosages.

Functionality and intolerance overlap: Numerous fermentable oligosaccharides, disaccharides, monosaccharides, and polyols (FODMAPs) function as prebiotic fibres (e.g., FOS, GOS, inulin), promoting intestinal health in those without IBS. Their elimination may diminish nutritional quality. FODMAPs affect dough rheology, fermentation processes, crumb texture, and product longevity. Minimizing them without compromising usefulness necessitates innovative methods (e.g., enzyme therapy, starting cultures with specific hydrolytic activity). To provide structure and nutrition to gluten-free products, legume flours and fibres, such as inulin, can unintentionally raise the FODMAP levels. To balance tolerance and quality, strategic component substitutions and fermentation processes are needed [87].

### 4.2. New and Established Approaches to Reduction

A biotechnological tool with great promise for reducing FODMAP levels in cereal-based baked goods is sourdough fermentation. The fermentation process for sourdough culture (*Lactobacilli* plus yeast) is gradual and carefully controlled, using a complex community of yeasts and lactic acid bacteria (LAB). Some oligosaccharides, such as fructans, which are a key component of wheat and rye products high in FODMAPs, may be broken down by these microorganisms [97]. Previous research indicates that sourdough fermentation can lower fructan levels by about 70–80%. However, a lot depends on the strain used, fermentation conditions (temperature, humidity, and duration), and the type of flour. *Lactobacillus paracasei*, *Lactobacillus plantarum*, and *Lactobacillus brevis* are examples of specific LAB that produce both extracellular and intracellular fructanases that hydrolyse β-(2 → 1)- and β-(2 → 6)-linked fructosyl residues [98]. These enzymes break down high-molecular-weight fructans into smaller, easier-to-ferment carbohydrates. When combined with metabolically compatible yeast species such as *Saccharomyces cerevisiae* or *Kazachstania humilis*, the released mono- and disaccharides are swiftly metabolized, reducing the buildup of intermediate fermentable sugars that may lead to gastrointestinal symptoms in sensitive individuals [99]. Targeted fermentations, involving the intentional introduction of specific microbial strains and controlled fermentation processes, enhance conventional sourdough methods. By selecting and utilizing strains with elevated fructanase activity and optimizing fermentation kinetics, bakers can consistently reduce FODMAPs while maintaining preferred technological and sensory characteristics [4]. Sourdough preparation significantly alters FODMAP levels while enhancing dough rheology, flavour complexity, mineral bioavailability through phytate breakdown, and shelf-life stability. From a functional and nutritional perspective, sourdough technology provides a practical, consumer-acceptable method for producing baked goods with lower FODMAP levels. Individuals with irritable bowel syndrome (IBS) can really benefit from these items for a better feeling when fewer FODMAPs are consumed [100]. Sourdough-based approaches, as opposed to those that replace additives, leverage natural microbial metabolism to meet customer demand for “clean-label” products [101]. One possibility for the future is precision fermentation, which enables the strategic creation of starter cultures specifically for FODMAP breakdown while maintaining product quality and is based on microbial genetics and metabolomics [102].

### 4.3. Enzymatic Techniques

Accurate techniques for lowering FODMAP concentrations in cereal-based systems while preserving product integrity have emerged using enzymes [103]. Breaking down oligo- and polysaccharides that cannot be digested is simplified by exogenous enzymes such as fructanases, inulinases, and β-galactosidases. Dough mixing, fermentation, or treating raw materials and additives before incorporation into formulations are all applications for these enzymes [104].

Fructanases and inulinases break down (21) links in fructans and inulin, making shorter-chain oligosaccharides, fructose, or glucose. The primary FODMAPs component in bread, fructan, can be significantly reduced through this hydrolysis in doughs made from wheat and rye. Similarly, by working on (16)-linked galactosyl residues, galactosidases liberate sucrose and galactose from RFOs, which are abundant in bean flours and soy-based products [105]. However, fermentable substrates are provided by monosaccharides, disaccharides, and occasionally polyols, the products of enzymatic degradation. If maintained in the matrix, these compounds may temporarily increase osmotic pressure in the gastrointestinal tract. Since individuals sensitive to FODMAPs may experience symptoms not from the parent oligosaccharide but from its breakdown products, this poses a significant challenge for formulation. To alleviate this danger, process design must incorporate enzyme activity into the fermentation stages. In sourdough systems, lactic acid bacteria and yeasts can metabolise the released sugars, lowering their concentration in the finished product [106]. The pH, amount of water in the dough, and the length of time the dough is mixed also affect how well enzymes work. This approach changes how enzymes function and how microorganisms use the products of hydrolysis. A suitable method for producing low-FODMAP baked goods without sacrificing bread and dough quality is the controlled application, typically carried out in steps or using a combination of enzymatic and fermentative techniques [107].

### 4.4. Raw Material Selection and Fractionation

A straightforward strategy to reduce FODMAP levels in bakery systems is to carefully select raw ingredients. Traditional baked goods primarily contain fructans because of the natural structure of cereal grains such as wheat and rye [106]. Utilizing intrinsic genotypic variability is the first step in implementing strategies to reduce this expense. A lot of research has shown that different wheat varieties have very different fructan levels. Low-fructan genotypes could be helpful in specialist breeding and ingredient production. Using these plants can significantly reduce the initial amount of fermentable carbohydrates without requiring significant process modifications [108]. Choosing the proper variety is another essential step, along with grain fractionation. Fructans are not evenly distributed throughout grain kernels; instead, they are more concentrated in the outer bran and aleurone layers [109].

Changes to the milling and sifting processes can be used to separate the fructan-rich parts from the starchy endosperm. That makes it easier to produce low-fructan flour streams suitable for bread-making. However, phytochemicals, micronutrients, and fiber levels decrease when bran fractions are removed [110]. Adding some low-FODMAP fibres, such as RS, beta-glucans, or psyllium husk, can help prevent nutrient loss [111]. Another method involves partial replacement with intrinsically low-FODMAP flours, such as rice, maize, sorghum, millet, or particular oat cultivars with reduced oligosaccharide levels. These flours improve the variety of nutrients and functions while also lowering the overall FODMAP load. Dough’s ability to be viscoelastic and hold gas, however, is hampered by lower gluten levels or an inability to form gluten [3]. To restore the volume, texture, and sensory aspects of bread, it often requires technical fixes, such as adding hydrocolloids (e.g., xanthan gum or hydroxypropyl methylcellulose) or modified starches [112]. When making low-FODMAP baked goods, these methods demonstrate that selecting the appropriate base materials and breaking them down into smaller pieces are also crucial steps. Gut microbiota implementation must strike a balance between reducing fermentable substrates, maintaining technical performance, and preserving nutritional and sensory characteristics to be successful [113].

### 4.5. High-Pressure Processing, Sprouting, and Other Process Innovations

New process innovations provide more options for adjusting FODMAP levels in baked products and cereals, in addition to selecting ingredients and fermentation [114]. These methods utilize biological or physicochemical modifications to diminish fermentable carbohydrate fractions while affecting product quality characteristics [115]. Cereal grain intrinsic enzyme systems, particularly fructan exohydrolases, invertases, and amylases, are activated during controlled sprouting, facilitating the hydrolysis of stored fructans and other carbohydrates into simple sugars. Studies show that germination can significantly reduce fructan levels in wheat, rye, and barley, with reductions of 30% to 70% depending on the duration, temperature, and moisture [116]. Sprouting not only lowers FODMAPs but also increases the availability of micronutrients (including iron, zinc, and folate), boosts antioxidant activity, and alters food taste. More free sugars from hydrolysis may influence sweetness and browning during baking, allowing for recipe adjustments [117].

Extrusion uses heat, shear forces, and pressure to shape materials. The intensity breaks down cell membranes, dissolves dietary fiber, and turns starch into a gel, which could change the ease of accessibility and the chemical stability of FODMAPs [118]. Extrusion of fructan-rich cereals has been documented to depolymerise fructans and diminish the quantifiable FODMAPs fraction partially. The extent of reduction depends on feed moisture, barrel temperature, and screw speed [119]. Additionally, extrusion can increase the levels of RS and retrograded starch fractions, which might affect glycemic response and possibly change how easily the colon ferments food. The texture of extruded flours affects water absorption and dough expansion. Therefore, it’s important to optimize the process to achieve the right balance of functional and nutritional outcomes [120].

High-pressure processing (HPP) applies uniform isostatic pressure (100–600 MPa) at ambient or moderate temperatures, leading to non-thermal structural modifications in biopolymers [121]. Pressure can change the shape of polymers, break weak hydrogen bonds, and alter the solubility of oligo- and polysaccharides [122]. HPP has also been shown to reduce the extractability of fructans and change the distribution of water in grain matrices. There could be lower quantities of FODMAPs that can be detected. Research by Antti Nyyssölä et al. [123] indicates that fermentation and enzymatic treatments can reduce FODMAP content by approximately 30–90%, depending on processing conditions and substrate composition. However, results vary according to water activity, holding time, and pressure level. Current research focuses on the effect on dough rheology and texture, revealing improved crumbs’ softness as well as detrimental effects on the integrity of the gluten network [124].

### 4.6. Nutritional Trade-Offs and Microbiome Implications

For individuals suffering from irritable bowel syndrome (IBS) and the gastrointestinal sensitivities that go along with it, cereal-based products that contain fewer FODMAPs have significant benefits [125]. Reducing consumption of fast-fermentable oligosaccharides, disaccharides, monosaccharides, and polyols can alleviate symptoms such as bloating, stomach pain, and diarrhea. Studies show that nutritional alterations entail trade-offs [103]. Numerous FODMAPs, particularly fructans and galacto-oligosaccharides (GOS), serve as prebiotics that specifically promote the growth of beneficial gut microbes such as *Lactobacillus* and *Bifidobacterium* species. Consequently, prolonged adherence to a rigorously low-FODMAP diet may unintentionally decrease the prevalence of these taxa. The compromise microbial diversity and the synthesis of short-chain fatty acids (SCFAs), particularly butyrate, which are crucial for gut and systemic health. Recent advancements in food processing aim to address this problem by carefully adjusting the fermentable carbohydrate content of baked goods [126]. Specific lactic acid bacteria can hydrolyze high-molecular-weight fructans during targeted sourdough fermentations. They contain RS and some soluble fibres, which serve as fermented substrates for the microbiota. Keeping substrates that help good fermentation processes will help lower FODMAPs that cause symptoms. In low-FODMAP formulations, different fibres like glucans, arabinoxylans, or new hydrocolloids may make up for the lack of prebiotic components in food [127].

The overall impact of these methods on host health depends on how microbial ecology and metabolic products interact [128]. In vitro fecal fermentation models provide initial data, facilitating the assessment of variations in gas production, microbial composition, and short-chain fatty acid profiles in response to modifications in cereal matrices. Human feeding trials are essential for translating these findings, as they encompass host-specific factors such as gastrointestinal transit, immunological modulation, and symptom response [129]. Low-FODMAP sourdough breads have been shown in recent clinical trials to alleviate gastrointestinal symptoms while maintaining or potentially expanding populations of butyrogenic taxa under certain conditions. Finding a balance between relieving symptoms and keeping the microbiome’s long-term health is the biggest challenge. Instead of eliminating fermentable carbohydrates, the best strategy is to selectively reduce the FODMAPs that cause symptoms and intentionally add or improve other prebiotic fibres [130]. This approach is consistent with precision nutrition, which customizes dietary interventions for symptom management and the maintenance of a robust, health-enhancing gut microbiota [131].

## 5. High-Quality FODMAPs Baked Products Utilizing Starch Derivatives and Polysaccharides Methods

Polysaccharides and modified starches can support digestive health while simultaneously improving technical quality without increasing sensitivity to FODMAP [132]. In dough systems, RS2, RS3, and RS4 add bulk, hold water, and soften the crumb. They also serve as fermentable substrates for colonic bacteria without causing the osmotic or fast fermentation issues associated with FODMAP. SCFAs, particularly butyrate, which are beneficial to colon health, are frequently produced through their selective fermentation. Likewise, several modified starches impart viscosity, freeze-thaw stability, or film-forming properties without adding to the fermentable carbohydrate reservoir that aggravates symptoms [133].

By imitating the viscoelastic properties of gluten or bran fructans, hydrocolloids like xanthan gum and hydroxypropyl methylcellulose (HPMC) enhance low-FODMAP baking [103]. They improve freeze-thaw stability, maintain the softness of the crumbs throughout storage, and stabilize gas cells during proofing and baking. Their non-fermentable or slowly fermentable properties are suitable for sensitive people, while preserving the highest possible product quality. Formulation strategies that strike a balance between technical efficacy and digestive tolerance can be developed more easily by combining hydrocolloids and RS [134].

On the other hand, some polysaccharides combine beneficial functional properties with negative FODMAP properties [135]. Inulin and short-chain fructooligosaccharides (FOS) provide a clear example. These substances are widely used for fiber enrichment, sugar substitution, fat imitation, and to develop creaminess and mouthfeel. These are appealing for both dairy and bread systems due to their technological characteristics. However, due to their quick fermentation in the intestines and vigorous osmotic activity, these are classified as high-FODMAP. They often cause symptoms in individuals sensitive to them [136]. Due to this discrepancy, conflict arises between innovative nutritional concepts, such as adding prebiotics or fiber at the right place, and how well these work in practice. Therefore, formulators must consider operational replacements such as RS, polydextrose, or -glucans, which provide bulk and ferment more slowly while reducing the risk of symptoms [137].

Process-based mitigation: Specific enzymatic hydrolysis (e.g., inulinases and fructanases) or fermentation procedures that break down FODMAP components while preserving their textural benefits are used during dough processing. Protective measures for sensory experiences: Ensuring that alternatives don’t alter the sweetness, creaminess, or crumb tenderness that inulin/FOS greatly improves [138].

Making low-FODMAP baked goods requires a planned method that balances gastrointestinal tolerance, product quality, and nutritional effectiveness. A variety of design heuristics can aid the formulation process. For instance, mapping out the FODMAP profiles of each component is a crucial initial step. To do this, consideration is needed of how much fructans, inulin, and galacto-oligosaccharides (GOS) are present in each raw material [139]. Enzymatic or chromatographic laboratory verification must be added to ingredient specification sheets to ensure precise quantification [140]. Finding specific substitutes or pre-treatment methods is made simpler by quickly identifying high-FODMAP components [141]. Choose polysaccharide blends carefully, as functional polysaccharides are essential for dough rheology, water retention, and crumb stabilization. Formulators may amalgamate starch derivatives (e.g., pregelatinized starches, modified starches) that confer paste stability, inhibit staling with neutral hydrocolloids (e.g., xanthan, HPMC, guar gum), and emulate the viscoelastic properties, which sometimes diminish upon the removal of fructan-rich components. These mixes help maintain volume, texture, and shelf life while reducing FODMAP levels.

Employ targeted fermentation or enzymatic steps: Controlled processing strategies can specifically diminish FODMAPs. Sourdough fermentation using fructan-hydrolysing lactic acid bacteria or the incorporation of exogenous enzymes (inulinases, α-galactosidases) might reduce undesirable fractions, according to [5]. Mechanisms must be adjusted to avert the excessive accumulation of simple sugars or polyols, which could replace one intolerance trigger with another. Decreasing or eliminating fructan- or GOS-rich fibres frequently reduces prebiotic efficacy. To restore functional benefits, RS or non-FODMAP soluble fibres (e.g., β-glucan, partially hydrolyzed guar gum, citrus pectin) may be included [142]. This replacement preserves dietary fiber levels and promotes gastrointestinal health without aggravating symptoms in sensitive individuals.

Several studies have confirmed that product development must not depend exclusively on formulation heuristics [143]. Though validation is necessary, encompassing (i) laboratory quantification of residual FODMAPs using HPLC or particular enzymatic assays, and (ii) in vitro gut fermentation investigations or in vivo clinical/cohort trials to determine tolerance and functional consequences, is imperative. Items are guaranteed to be both technically sound and clinically relevant by this multi-tier validation.

## 6. Analytical and Methodological Considerations

Low-FODMAP baked goods require stringent analytical and methodological frameworks to guarantee compositional accuracy and functional relevance [144]. For regulatory compliance and consumer safety, it is essential to measure FODMAPs accurately. Validated chromatographic and enzymatic assays are required for some fractions, such as fructans, galacto-oligosaccharides (GOS), lactose, and polyols. Food processing, especially fermentation, can alter the FODMAPs profile in real time by converting one substrate into another. For example, fructan hydrolysis changes fructose or glucose into free fructose or glucose [145]. To capture these dynamic changes, time-resolved or process-integrated monitoring is more essential than static endpoint measurements.

In addition to compositional analysis, it is imperative to systematically combine structural and sensory assessments. Extensograph testing and other modern rheological instruments, such as Dynamic Mechanical Thermal Analysis (DMTA), make it easier to obtain reliable measurements of dough’s extensibility and viscoelasticity during processing [146]. Microstructural imaging techniques, including confocal laser scanning microscopy and scanning electron microscopy (SEM), provide supplementary insights into polymer interactions and gas-cell design. Combining instrumental data with sensory panel ratings demonstrates how molecular changes can affect consumer-relevant characteristics such as mouthfeel, volume, and texture.

The primary aim of FODMAP reduction is to improve gastrointestinal tolerance; however, unintended nutritional deficiencies may arise if fermentable substrates essential for optimal gut microbiota are removed. In vitro fecal fermentation systems provide a controlled setting for assessing microbial uptake of reformulated products [147]. Combining metabolomic analysis of SCFAs with targeted sequencing methods (16S rRNA or metagenomics) makes it easier to evaluate changes in microbial communities and the functional metabolic products they produce. Recent investigations utilizing these integrative approaches underscore the strain-dependent characteristics of fructans degradation, demonstrating varied downstream impacts on microbiota composition and metabolite synthesis. These results emphasize the significance of evaluating low-FODMAP formulations for clinical tolerability and their potential to sustain a healthy gut microbiome [148].

## 7. Knowledge Gaps and Future Research Directions

Notwithstanding significant advancements in understanding the function of polysaccharides and the regulation of FODMAPs in biological systems, some essential deficiencies persist. Food chemistry, process engineering, microbiology, and clinical nutrition must all work together to solve these problems. Existing understanding establishes qualitative correlations among polysaccharide type, rheological behaviour, and sensory results, although it lacks mechanistic specificity [149]. An urgent requirement exists for predictive rheological models that explicitly include polymer chain topology, branching configurations, hydration dynamics, and protein–polysaccharide interactions. Modeling is challenging due to: (i) Structural heterogeneity of polysaccharides. (ii) Variability in molecular weight and branching. (iii) Complex, nonlinear viscoelastic behavior. (iv) Multiscale interactions between polymers, water, and other food components [150]. These models would facilitate virtual screening of hydrocolloid mixtures and modified starches, diminishing dependence on expensive trial-and-error formulation [151].

Enzymatic and microbiological therapies show potential for specific FODMAP reduction; however, regulation of breakdown pathways remains inadequate [152]. The directed evolution or rational selection of carbohydrate-active enzymes (CAZymes) and lactic acid bacteria (LAB) strains that can selectively degrade fructans, galactooligosaccharides, or polyols without producing symptom-inducing intermediates is a goal. Recent analyses of sourdough starter strains indicate potential viability; however, the relationship between strain, substrate, and process is inadequately delineated, necessitating comprehensive metabolic and process-level characterisation [153].

The majority of research in this domain focuses on chemical measurement of FODMAPs or on the technological efficacy of formulations [118]. Data linking formulation or processing advances to significant improvements in gastrointestinal tolerance are limited [114]. To confirm that low-FODMAPs baking methods provide substantial consumer benefits while maintaining long-term gut health, standardized clinical objectives, such as symptom evaluation, gastrointestinal transit markers, and microbiota variation, are essential. Physical changes, including annealing, extrusion, and high-pressure processing, can alter the functionality of starches and hydrocolloids while reducing FODMAPs content, making them sustainable and scalable approaches for tailoring starch and hydrocolloid properties [10,154]. These support the creation of gut-friendly, low-FODMAP, and health-oriented food products and align with sustainable food engineering.

## 8. Conclusions

Recent advances in starch polymer science and glycoscience have enabled the rational design of baked goods with tailored functionality, driven by a deeper understanding of structure function relationships in starch systems. A central mechanism underpinning these developments is the precise modulation of starch architecture, particularly amylose–amylopectin ratio and branch-chain distribution. Enzymatic tools such as amylases and transglycosidases catalyse α-(1 → 4) and α-(1 → 6) glycosidic bond cleavage, transfer, and re-branching, generating structured starches with controlled molecular weight and functionality. These molecular-level modifications, combined with green physical treatments (e.g., heat–moisture treatment, extrusion, and pressure cycling), alter starch crystallinity and gelatinisation behaviour, thereby enabling fine-tuning of dough rheology, gas retention, and crumb microstructure.

A major finding of this work is that glycoscience-driven enzymatic and fermentation strategies enable substantial FODMAP reduction without compromising product quality. Mechanistically, enzymes such as α-galactosidase and fructanase selectively hydrolyse galacto-oligosaccharides and fructans into digestible sugars, while targeted sourdough fermentations employing specialized yeasts and lactic acid bacteria achieve 80–90% degradation of fermentable carbohydrates. Notably, engineered strains (e.g., *Kluyveromyces marxianus*) further enhance fructan hydrolysis (>90%), demonstrating the potential of microbial biocatalysis as a precision tool in bakery applications. Importantly, these interventions preserve critical quality attributes, including loaf volume, crumb structure, and sensory properties.

Another key outcome is the simultaneous enhancement of nutritional quality through bioprocessing mechanisms. Extended fermentation and enzymatic dephytinization degrade phytic acid, releasing bound minerals and significantly improving bioavailability. For example, sourdough fermentation can eliminate phytate and increase iron absorption by up to 3.5-fold, highlighting the dual functionality of these processes in improving both digestibility and nutritional value. Looking ahead, integrating advanced analytical techniques (e.g., differential scanning calorimetry, rheometry, spectroscopy) with microbiome-guided fermentation design represents a critical pathway for next-generation bakery innovation. These approaches will enable predictive control of starch functionality and the optimization of microbial consortia tailored to specific formulations. Collectively, the convergence of polymer science, enzymology, and fermentation technology establishes a robust framework for developing low-FODMAP, nutritionally enhanced, and consumer-acceptable baked products.

Ultimately, this work demonstrates that targeted manipulation of starch molecular structure, combined with enzymatic and microbial processing, is the key mechanism linking carbohydrate chemistry to baking performance and health outcomes, bridging fundamental glycoscience with practical food innovation.

## Figures and Tables

**Table 1:** Starch modification methods.

Modification Type	Techniques	Structural Impact	Baking/Food Relevance	Example	Reference
Physical	HMT, annealing, PR gelatinization, extrusion, ultrasound	Alters crystallinity, granule integrity, and swelling	Improves dough handling, shelf life, and cold-water solubility	Pregelatinized wheat starch in cake mixes	[26]
Chemical	Cross-linking, acetylation, hydroxypropylation, oxidation	Alters crystallinity, granule integrity, and swelling	High stability under shear/heat; anti-staling; freeze-thaw resistant	Cross-linked starch in frozen doughs	[14]
Enzymatic	Amylases, pullulanase, BE, CGTase	Hydrolysis or re-branching produces oligosaccharides or RS	Tailored digestibility, crumb structure, and prebiotic potential	Debranched starch for RS3-rich breads	[27]

**Table 3:** Overall interpretation.

Parameter	Improves with	Deteriorates with	Meaning	Reference
Stickiness	Less HPMC	More Xanthan	HPMC makes dough easier to handle	[74]
Firmness	More HPMC	More Xanthan	HPMC softens crumb	[73]
Specific volume	More HPMC	More Xanthan	HPMC helps bread rise	[79]
Baking loss	Less Xanthan	More Xanthan	HPMC retains moisture	[79]
Water activity	More Xanthan	More HPMC	HPMC improves storage stability	[79]
Crust L*	More HPMC	More Xanthan	HPMC yields a lighter crust	[75]
Crumb L*	Balanced mix	Excess Xanthan	Proper blend yields lighter, softer crumb	[80]

## Data Availability

The data presented in this study are available on request from the corresponding author due to (specify the reason for the restriction).

## Abbreviations

Abbreviation	Definition
FODMAP	fermentable oligosaccharides, disaccharides, monosaccharides, and polyols
LAB	lactic acid bacteria
SCFAs	short-chain fatty acids
IBS	Irritable bowel syndrome
RS	resistant starch
CMC	carboxymethyl cellulose
OSA	Octenyl succinic anhydride
HMT	Heat–moisture treatment
MCR	Modular Compact Rheometer
HPMC	hydroxypropyl methylcellulose
SEM	scanning electron microscopy
DMTA	Dynamic Mechanical Thermal Analysis
